# The Age-Specific Quantitative Effects of Metabolic Risk Factors on Cardiovascular Diseases and Diabetes: A Pooled Analysis

**DOI:** 10.1371/journal.pone.0065174

**Published:** 2013-07-30

**Authors:** Gitanjali M. Singh, Goodarz Danaei, Farshad Farzadfar, Gretchen A. Stevens, Mark Woodward, David Wormser, Stephen Kaptoge, Gary Whitlock, Qing Qiao, Sarah Lewington, Emanuele Di Angelantonio, Stephen vander Hoorn, Carlene M. M. Lawes, Mohammed K. Ali, Dariush Mozaffarian, Majid Ezzati, Global Burden of Metabolic Risk Factors of Chronic Diseases Collaborating Group; Asia-Pacific Cohort Studies Collaboration (APCSC), Diabetes Epidemiology: Collaborative analysis of Diagnostic criteria in Europe (DECODE), Emerging Risk Factor Collaboration (ERFC), Prospective Studies Collaboration (PSC)

**Affiliations:** 1 Department of Nutrition, Harvard School of Public Health, Boston, Massachusetts, United States of America; 2 Department of Global Health and Population, Harvard School of Public Health, Boston, Massachusetts, United States of America; 3 Department of Epidemiology, Harvard School of Public Health, Boston, Massachusetts, United States of America; 4 Diabetes Research Centre and Endocrinology and Metabolism Research Centre, Tehran University of Medical Sciences, Tehran, Iran; 5 Department of Health Statistics and Information Systems, World Health Organization, Geneva, Switzerland; 6 The George Institute for Global Health, University of Sydney, Australia; 7 Department of Epidemiology, Johns Hopkins University, Baltimore, Maryland, United States of America; 8 Department of Public Health and Primary Care, University of Cambridge, Cambridge, United Kingdom; 9 Clinical Trial Service Unit, University of Oxford, Oxford, United Kingdom; 10 Department of Public Health, University of Helsinki, Helsinki, Finland; 11 Department of Statistics, University of Melbourne, Melbourne, Australia; 12 National Institute for Health Innovation, University of Auckland, Auckland, New Zealand; 13 Hubert Department of Global Health, Emory University, Atlanta, Georgia, United States of America; 14 Division of Cardiovascular Medicine and Channing Laboratory, Department of Medicine, Brigham and Women's Hospital and Harvard Medical School, Boston, Massachusetts, United States of America; 15 Medical Research Council-Health Protection Agency Centre for Environment and Health, Department of Epidemiology and Biostatistics, School of Public Health, Imperial College London, London, UK; John Hopkins Bloomberg School of Public Health, United States of America

## Abstract

**Background:**

The effects of systolic blood pressure (SBP), serum total cholesterol (TC), fasting plasma glucose (FPG), and body mass index (BMI) on the risk of cardiovascular diseases (CVD) have been established in epidemiological studies, but consistent estimates of effect sizes by age and sex are not available.

**Methods:**

We reviewed large cohort pooling projects, evaluating effects of baseline or usual exposure to metabolic risks on ischemic heart disease (IHD), hypertensive heart disease (HHD), stroke, diabetes, and, as relevant selected other CVDs, after adjusting for important confounders. We pooled all data to estimate relative risks (RRs) for each risk factor and examined effect modification by age or other factors, using random effects models.

**Results:**

Across all risk factors, an average of 123 cohorts provided data on 1.4 million individuals and 52,000 CVD events. Each metabolic risk factor was robustly related to CVD. At the baseline age of 55–64 years, the RR for 10 mmHg higher SBP was largest for HHD (2.16; 95% CI 2.09–2.24), followed by effects on both stroke subtypes (1.66; 1.39–1.98 for hemorrhagic stroke and 1.63; 1.57–1.69 for ischemic stroke). In the same age group, RRs for 1 mmol/L higher TC were 1.44 (1.29–1.61) for IHD and 1.20 (1.15–1.25) for ischemic stroke. The RRs for 5 kg/m^2^ higher BMI for ages 55–64 ranged from 2.32 (2.04–2.63) for diabetes, to 1.44 (1.40–1.48) for IHD. For 1 mmol/L higher FPG, RRs in this age group were 1.18 (1.08–1.29) for IHD and 1.14 (1.01–1.29) for total stroke. For all risk factors, proportional effects declined with age, were generally consistent by sex, and differed by region in only a few age groups for certain risk factor-disease pairs.

**Conclusion:**

Our results provide robust, comparable and precise estimates of the effects of major metabolic risk factors on CVD and diabetes by age group.

## Introduction

Globally, roughly 17 million deaths are caused by cardiovascular disease (CVD) and diabetes each year [1]. Although the major metabolic risk factors for these diseases have been characterized in epidemiological studies, consistent measurements of their effects by age, sex and region are not available. Understanding the effects of metabolic risk factors on CVD mortality and burden of disease are important inputs for policy and priority setting related to disease prevention.

Population-based risk assessment requires data on population exposure to risk factors and on the magnitude of their effects on different disease outcomes [2], [3]. Effect estimates in prior global comparative risk assessment (CRA) analyses of metabolic risk factors including systolic blood pressure (SBP), serum total cholesterol (TC), fasting plasma glucose (FPG), and body mass index (BMI) were based on the Asia Pacific Cohort Studies Collaboration (APCSC) and selected other cohort pooling studies [3]–[13]. Since that time, several additional meta-analyses have become available for Western and Asian populations [14]–[22]. There is, however, no systematic evaluation and comparison of these sources for new global and national risk assessments, including potential heterogeneity by age, sex, or region. The aim of this study was to provide robust, comparable, and consistent effects of major metabolic risk factors on CVD and diabetes, including variation in these effects by age, sex, or region.

## Methods

### Metabolic risk factors

We compared and pooled RRs for the effects of key metabolic risk factors: SBP, TC, FPG, and adiposity measured by BMI, from major global pooling projects. For SBP, TC, and FPG, we focused on the usual distribution, i.e., the distribution that has been corrected for temporal changes in measurement over time (such data were not available for BMI; see also below). The choice of exposure metrics was based on their associations with disease outcomes and on the availability of worldwide exposure data in previously described systematic analyses [23]–[26]. In particular, we do not present results for other related risk factors such as low-density lipoprotein (LDL) cholesterol, Hemoglobin A1c, waist circumference, and waist-to-hip ratio, because global exposure data to subsequently quantify effects on disease burdens are significantly more limited [24]–[26].

### Data sources

To obtain RR per unit of exposure for diseases with probable or convincing etiologic associations with each risk factor, we used existing meta-analyses of epidemiological studies. We selected large comprehensive pooling projects of observational studies that estimated the effects of baseline or usual exposure for the risk factors and outcomes of interest by age group. Even when randomized studies were available, we used observational studies because (i) they estimate the effect of risk factor levels on disease outcome as opposed to the effect of a particular pharmacological intervention which may act through risk factor reduction as well as other pathways, (ii) they estimate the long-term effects (over years or decades) of exposure to risk factors as opposed to effect of short-term changes due to treatment in randomized trials, and (iii) they generally have larger sample sizes and can provide more precise RRs for more detailed age groups and disease categories. Randomized trials were used to support the evidence on the presence of causal effects from observational studies.

The sources used were the Asia Pacific Cohort Studies Collaboration (APCSC), the Diabetes Epidemiology: Collaborative analysis of Diagnostic criteria in Europe (DECODE), the Emerging Risk Factor Collaboration (ERFC) and the Prospective Studies Collaboration (PSC) [9]–[13], [16]–[22], [27]–[29]. All four cohort pooling studies have large numbers of participants and events that allow the estimation of RRs by age and disease outcome. Further, all these pooling studies used individual level data which allows for more consistent adjustment for confounders. PSC included only fatal events, while APCSC and ERFC included both fatal and non-fatal events. ERFC and PSC excluded participants with pre-existing vascular disease, while APCSC did not. Some cohorts were included in both multiple pooling studies but the overlaps were relatively small. We therefore used results from all of the above-mentioned pooling projects when available. All pooling studies included in this analysis adjusted for age and sex, and accounted for differences in risk by cohort. We did not use the National Cancer Institute (NCI) Cohort Consortium [14] and a recent pooled analysis of Asian cohorts [15] for BMI effect sizes because these studies reported RRs for cardiovascular diseases (CVD) combined but not separately for ischemic heart disease (IHD) and stroke. Most data are from published sources but, when possible, re-analyses were done to obtain RRs for diseases and age groups of interest.

When possible, we used RRs for SBP, TC and FPG that were adjusted for regression dilution bias using repeated exposure measurements. Evidence from a large prospective study with multiple measurements of weight and height showed that regression dilution bias did not substantially affect the RRs for BMI, reflecting its relative stability compared with the other metabolic risk factors [30]. In their published results, the pooling studies used different methods for correcting regression dilution bias: e.g., PSC used age-specific correction factors and also accounted for time between baseline risk factor measurements and the occurrence of events. We conducted a re-analysis of APCSC for SBP to use consistent approaches with the PSC meta-analysis. DECODE and ERFC did not adjust for regression dilution bias.

### Disease outcomes

The disease outcomes included in this analysis were: ischemic heart disease (IHD) (ICD-10 codes I20–I25), ischemic stroke (I63, I65–I67, I69.3), hemorrhagic stroke (I60-62, I69.0-2), hypertensive heart disease (HHD) (I11–I13), aortic aneurysm (I71), rheumatic heart disease (RHD) (I01, I02.0, I05–I09), inflammatory heart disease (I33, I42), and diabetes (E10–E14). Cardiovascular outcomes reported in the pooling projects other than those reported above were included in the category “Other cardiovascular disease”. Outcomes were adjudicated by medical authorities within each cohort.

Table 1 presents the selected risk factors and the disease outcomes affected by each risk factor using evidence from observational studies, supported by randomized trials when available and applicable [31]–[33]. Specifically, randomized trials have shown that reducing blood pressure lowers the risk of mortality from heart failure [32], [33], which is considered an intermediate, vs. underlying, cause of death [34]. Therefore, we included cardiovascular diseases that lead to heart failure such as rheumatic heart disease as outcomes for SBP, but the reported RRs should only be applied to mortality from these causes (as opposed to incidence) because the incidence of diseases like RHD and other inflammatory heart diseases is unlikely to be affected by SBP.

**Table 1:** Outcomes associated with each risk factor, studies from which RRs were extracted, and procedures for estimating RRs by age group.

Disease outcome	Studies that reported RRs	Procedures for estimating RRs in standardized age groups
**Systolic blood pressure (SBP)**		
Ischemic heart disease (IHD)	PSC	Interpolation and extrapolation
	APCSC	Interpolation and extrapolation
Ischaemic stroke	PSC	Interpolation and extrapolation
	APCSC	Interpolation and extrapolation
Haemorrhagic stroke	PSC	Interpolation and extrapolation
	APCSC	Interpolation and extrapolation
Hypertensive heart disease	PSC	Redistribution from a single age group using PSC “SBP-other vascular diseases” age pattern
	APCSC	Interpolation and extrapolation
Rheumatic heart disease^a^	PSC	Redistribution from a single age group using PSC SBP-“other vascular diseases” age pattern
Inflammatory heart disease^a^	PSC	Redistribution from a single age group using PSC “SBP-other vascular diseases” age pattern
Aortic aneurysm	PSC	Redistribution from a single age group using PSC “SBP-other vascular diseases” age pattern
All other cardiovascular diseases^b^	PSC	Redistribution from a single age group using PSC “SBP-other vascular diseases” age pattern
	APCSC	Redistribution from a single age group using PSC “SBP-other vascular diseases” age pattern
**Total cholesterol (TC)**		
IHD	PSC^c^	Interpolation and extrapolation
	APCSC	Data provided in GBD age groups
Ischemic stroke	PSC	Interpolation and extrapolation
	APCSC	Data provided in GBD age groups
**Fasting plasma glucose (FPG)**		
IHD	DECODE	Interpolation and extrapolation
	APCSC	Interpolation and extrapolation
	ERFC	Interpolation and extrapolation.
Total stroke	DECODE	Interpolation and extrapolation
	APCSC	Interpolation and extrapolation
	ERFC	Interpolation and extrapolation
**Body mass index (BMI)**		
IHD	PSC	Interpolation and extrapolation
	APCSC	Interpolation and extrapolation
	ERFC	Interpolation and extrapolation
Ischaemic stroke	PSC	Redistribution from a single age group using PSC BMI-total stroke age pattern
	APCSC	Interpolation and extrapolation
	ERFC	Interpolation and extrapolation
Haemorrhagic stroke	PSC	Redistribution from a single age group using PSC BMI-total stroke age pattern
	ERFC	Interpolation and extrapolation
Diabetes	PSC	Interpolation and extrapolation
	APCSC	Interpolation and extrapolation

a RRs apply to mortality but not to incidence and is included because of the benefits of lower blood pressure for reduced heart failure mortality.

b This residual category contains a number of ICD codes. The proportion of deaths from the constituent diseases is likely to vary across world regions and even across cohorts in the same meta-analysis.

c A quadratic age model was used instead of a log-linear age model as this fit the data better.

### RRs by sex and age

We used RRs for both sexes combined because results of the included meta-analyses had shown that RRs between men and women were similar [10]–[13], [20]–[22]. Based on prior evidence that proportional effects of some metabolic risk factors vary by age, a key aim was to establish quantitative estimates of interaction by age. We estimated RRs for the following age groups: 25–34, 35–44, 45–54, 55–64, 65–74, 75–84 and 85+ years. We used the following approach to obtain RRs by the selected age groups from different sources (Table 1):

- When RRs were provided by age groups that differed from the above groups, we used interpolation to obtain RRs by the selected age groups.
- When RRs were provided for a narrower age range, we used extrapolation to estimate RRs for younger and/or older age groups.
- When RRs were provided for all ages combined, we re-analysed original data when these were accessible to the authors. If re-analysis was not possible, we assigned the single RR to the median age at event and used the age-association of the most similar risk factor-disease pair to estimate RRs by the selected age groups (redistribution).

Our interpolation and extrapolations used a linear relationship between ln(RR) and midpoint of age in each age category. This model had the best fit among a range of models including linear, quadratic, and cubic relationships between age and RR or ln(RR). The procedures used for different risk factor-disease pairs are reported in Table 1.

### Uncertainty of RRs

The uncertainty of the estimates of RRs has two components: (1) the (sampling) uncertainty of the RRs in the original source and (2) the uncertainty associated with conversion to age-specific RRs as outlined above. To estimate the overall uncertainty, we used a statistical simulation approach: in each of the 1,000 iterations, we drew a ln(RR) for each age group in the published meta-analyses from a normal distribution characterized by the reported ln(RR) and its standard error. We fitted a linear model to this set of age-specific ln(RR)s, and used the fitted model to estimate an RR for each selected age group. The distributions of the 1,000 estimated ln(RR)s were used to obtain the standard errors of the ln(RR)s in the selected age groups. We then pooled the age-specific RRs from multiple sources using a random effects model (meta.summaries command in the open-source statistical software R version 2.11.1).

### Theoretical minimum-risk exposure distribution (TMRED)

An additional input required for risk assessment is an alternative exposure distribution relative to which the effects of risk factors are measured. The theoretical minimum-risk exposure distribution (TMRED) is an alternative exposure distribution that aims to measure the effects of all non-optimal levels of exposure in a comparable way across risk factors [2], [3], [35], [36]. TMRED is the distribution that corresponds to the lowest risk of all-cause mortality. Since all metabolic indicators are necessary to sustain life, their ‘exposure-response’ relationship is J-shaped or U-shaped, i.e. there could be increased risk of adverse outcomes below some levels [18], [22]. However, the subjects in epidemiological studies often have exposures that do not allow reliable estimation of optimally low levels, i.e. where benefits stop and harms begin. For example, many Western cohorts include fewer subjects with low BMI levels; similarly, SBP levels at which the dose-response relationship with CVD may flatten or reverse seem to be below those seen in most epidemiological studies. As a result, to select TMREDs for the risk factors of interest, we used both the evidence from epidemiological studies with the levels of exposure observed in populations that are considered low-risk, e.g. populations that consume low salt for blood pressure and those that consume low animal fat diets for serum cholesterol [5], [6]. Specifically, we selected TMREDs as the lowest levels observed in observational, and when relevant randomised, epidemiological studies as long as the selected level was also seen at the population level regardless of age or sex. We used the same TMRED for both sexes and all age groups because the associations of metabolic risk factors with age are relatively flat in low-exposure populations [37], [38].

In addition to an empirically-based mean, the TMRED may also have a standard deviation (SD), on the premise that even in the absence of major environmental risk factors, there is some residual variation in metabolic risk factors in the population. Empirically, the SD of metabolic risk factors tends to be smaller in populations that have a lower mean, with an approximately linear relationship [6]. We used this relationship to estimate the SD of TMRED once its mean was established.

## Results

Across all risk factors, an average of 123 cohorts provided data on 1.42 million individuals having 52,000 CVD events. A total of 99 cohorts with 1.38 million participants and 65,000 CVD events informed the RRs for SBP. For TC, 1.2 million participants having 59,000 CVD events from 92 cohorts provided data for this analysis. BMI was the risk factor with effect estimates based on the largest number amount of data: 163 cohorts with 2.43 million participants and 70,000 CVD events. Of the four metabolic risk factors, FPG RRs were based on the fewest events, 7,000 events among 372,000 participants in 116 cohorts.

**Figure 1:**
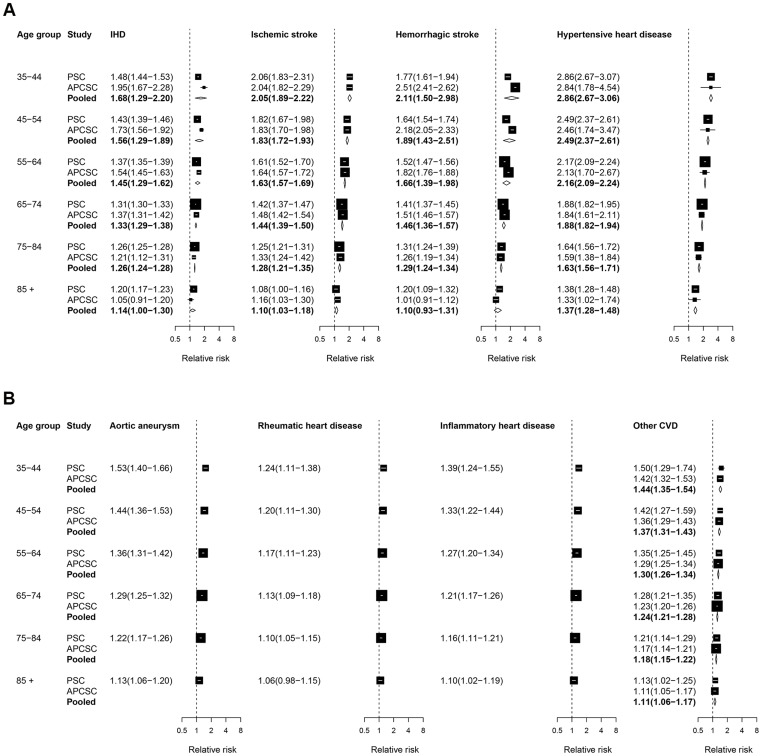
Relative risks (RRs) for diseases associated with systolic blood pressure (SBP). The figure shows RRs for 10 mmHg higher usual SBP. The figure shows RRs converted to comparable age group as described in Methods. See Table S1 for RRs in original age groups from each study. RRs for rheumatic heart disease and inflammatory heart disease apply only to deaths and those for other outcomes to deaths and incidence. The percentage of variation in the pooled estimates that is due to statistical heterogeneity was evaluated using the I^2^ statistic for each age group and outcome. Of all outcomes and age groups analyzed, only two age groups in the pooled analysis for hemorrhagic stroke had non-zero I^2^ values: I^2^ = 44.4% for ages 35–44 years, and I^2^ = 24.3% for ages 55–64 years.

Figure 1 presents the forest plot for the estimated effects of SBP on CVD outcomes. When age-specific RRs were available, we observed a clear age gradient, with smaller RRs in older ages. At a baseline age group of 55–64, the RR for SBP was largest for hypertensive heart disease, showing a more than doubling of the risk of this disease for each 10 mmHg higher SBP (2.16; 95% CI 2.09–2.24); this was followed by the effects on both stroke subtypes which had a two thirds increase in risk (1.66; 1.39–1.98 for haemorrhagic stroke and 1.63; 1.57–1.69 for ischemic stroke); it was smallest for rheumatic heart disease (1.17; 1.11–1.23).

**Figure 2:**
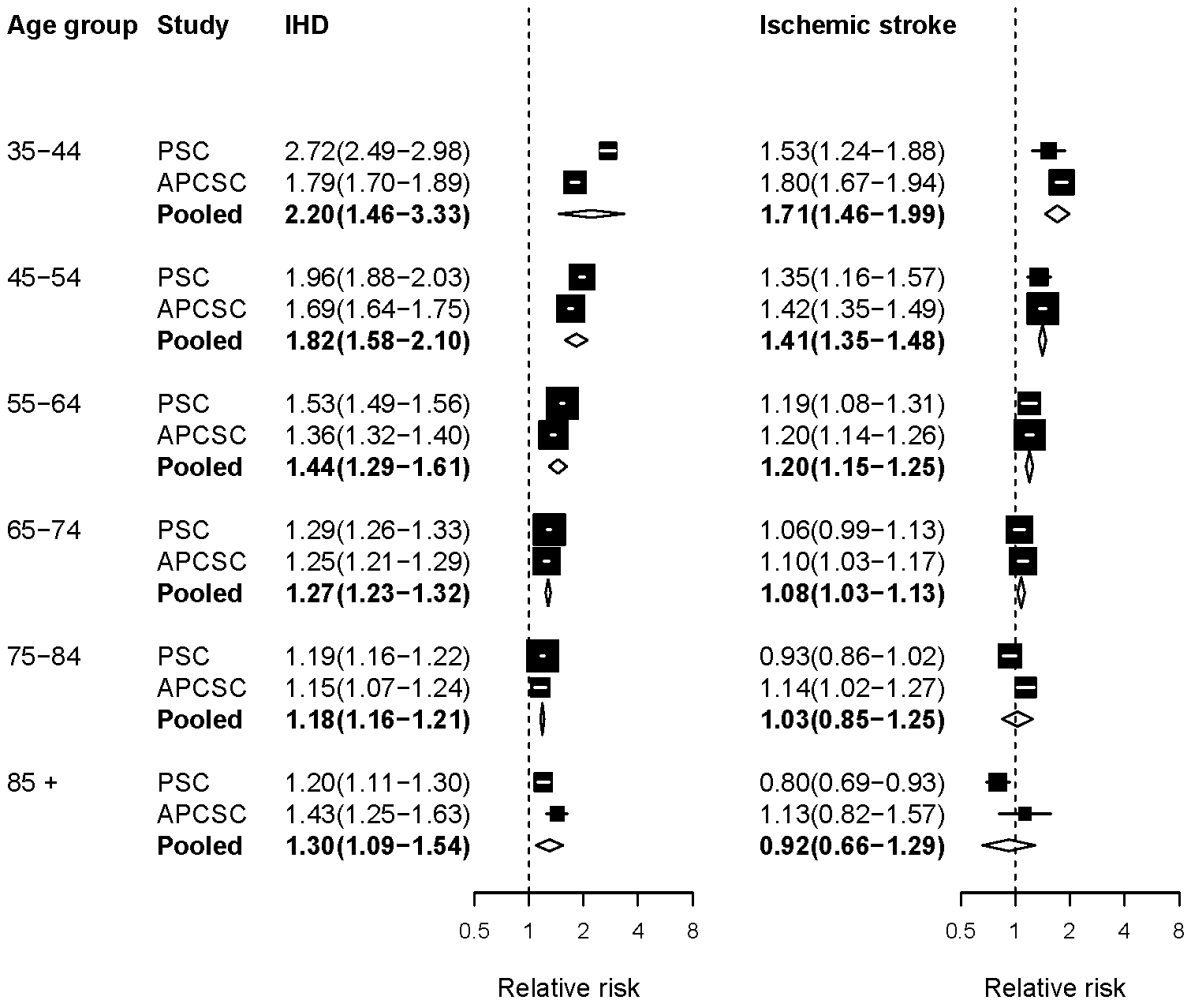
Relative risks (RRs) for diseases associated with serum total cholesterol (TC). The figure shows RRs for 1 mmol/L higher usual TC. The figure shows RRs converted to comparable age group as described in Methods. See Table S1 for RRs in original age groups from each study. The percentage of variation in the pooled estimates that is due to statistical heterogeneity was evaluated using the I^2^ statistic for each age group and outcome. Of all the outcomes and age groups analyzed, only ages 35–44 years in the pooled analysis for IHD had a non-zero I^2^ value of 58.8%.

The results from pooling two meta-analyses that reported RRs for TC are presented in Figure 2. The RRs were consistent across PSC and APCSC, except for the estimated effect of TC on IHD in those younger than 55 years of age, which was larger in PSC. There was a reduction in the RRs of IHD and ischaemic stroke with increasing age, similar to that seen for SBP. Indeed, the 95% confidence interval of the pooled RR included the null effect for ischaemic stroke in ages 75 years and older. There is evidence, from randomized trials of statins, that lowering serum cholesterol in participants with high CVD risk may lower the risk of stroke in those aged 70 years and older [39], [40]. However, this effect may be mediated through pathways other than lipid lowering, e.g., atheromatous plaque stabilization anti-inflammatory effects, or inhibition of platelet aggregation [41].

**Figure 3:**
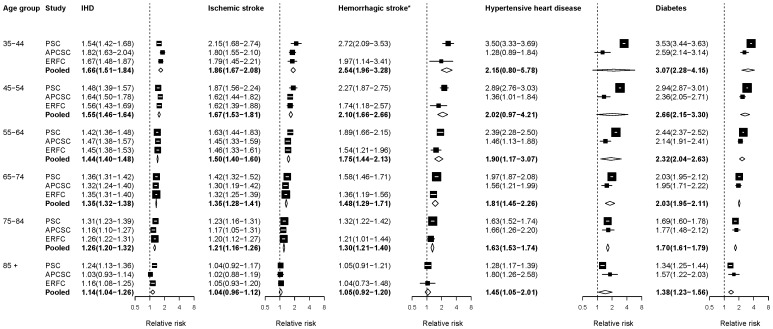
Relative risks (RRs) for diseases associated with body mass index (BMI). The figure shows RRs for 5 kg/m^2^ higher baseline BMI. The figure shows RRs converted to comparable age group as described in Methods. See Table S1 for RRs in original age groups from each study. The percentage of variation in the pooled estimates that is due to statistical heterogeneity was evaluated using the I^2^ statistic for each age group and outcome. Of all the outcomes and age groups analyzed, the three age groups below age 65 years in the pooled analysis for hypertensive heart disease had non-zero I^2^ values: 79.2% for ages 35–44 years, 69.0% for ages 45–54 years, and 37.2% for ages 55–64 years. *The associations with haemorrhagic stroke are for BMIs above 25 kg/m^2^ as described in text.

Figure 3 summarizes the RR estimates for the associations of BMI with CVD and diabetes. RRs for the estimated effect of BMI on diabetes and hypertensive heart disease were larger in Western cohorts as compared with Asian cohorts in adults <55 years old, perhaps due to longer exposure to high BMI in Western populations. Because there was no association between BMI and haemorrhagic stroke for BMIs up to 25 kg/m^2^ in APCSC, ERFC and PSC, we report the RR per unit of BMI above 25 kg/m^2^ pooled from these two meta-analyses.

**Figure 4:**
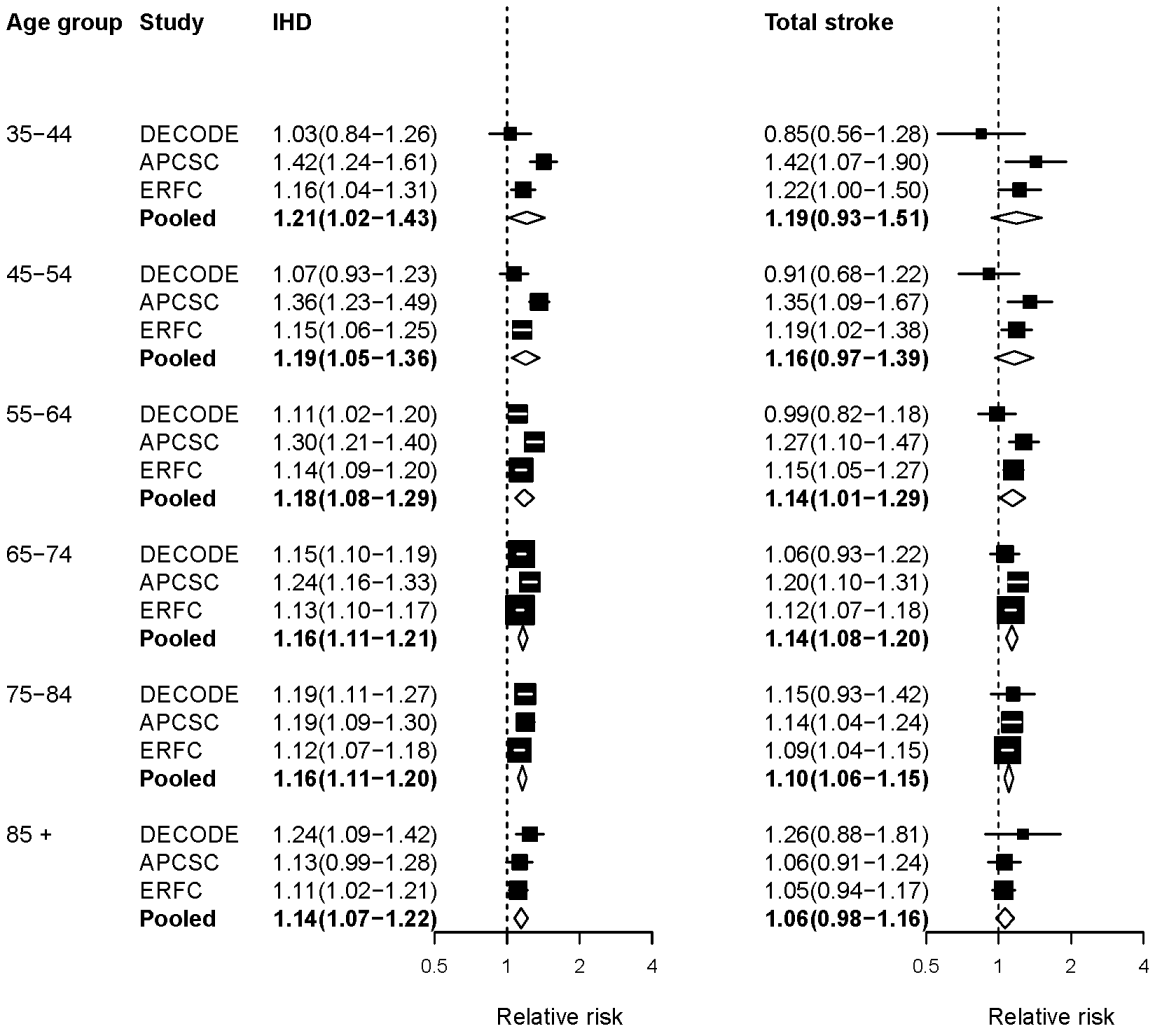
Relative risks (RRs) for diseases associated with fasting plasma glucose (FPG). The figure shows RRs for 1 mmol/L higher usual or baseline FPG. The figure shows RRs converted to comparable age group as described in Methods. See Table S1 for RRs in original age groups from each study. The percentage of variation in the pooled estimates that is due to statistical heterogeneity was evaluated using the I^2^ statistic for each age group and outcome. All I^2^ values for these outcomes and age groups were zero.

Figure 4 presents the forest plots for RRs per unit of FPG from 3 pooling studies. APCSC and ERFC did not report effects for subtypes of stroke separately, so we used RRs for stroke subtypes combined. Like the other metabolic risks, RRs declined with increasing age. The association between FPG and stroke was not statistically significant up to 55 years of age, due to non-significant protective effects in the DECODE study.

### Theoretical minimum-risk exposure distributions (TMREDs)

In previous CRA analyses, the mean(SD) of the TMREDs for metabolic risks were as follows: SBP 115(6) mmHg; TC 3.8(0.6) mmol/L; BMI 21(1) kg/m^2^; and FPG 4.9(0.3) mmol/L. More recent evidence from randomized trials of antihypertensive drugs suggests that benefits of lowering blood pressure may continue to 110 mmHg or lower [32]; the lowest observed levels in the populations included in the Intersalt study were below 100 mmHg [42]. For cholesterol, while Asian cohorts had subjects with TC levels below 4.0 mmol/L and estimated associations to as low as 3.8 mmol/L for IHD [11], [43], other epidemiological studies report mean levels of 4.0 mmol/L or more [8]. There may also be an increased risk of haemorrhagic stroke at low cholesterol levels [11],[44], [45]. Therefore we selected TMRED based on the low levels observed in observational studies and did not use randomized trials of statins because, as described earlier, statins may exert protective effects through pathways other than lowering cholesterol.

The observed rise in mortality at lower BMI levels may in some cases be due to ‘reverse causality’, as weight loss may precede death by a decade or more for many chronic diseases, particularly respiratory diseases and cancer. This phenomenon is also reflected in a more consistent dose-response relationship when analyses are restricted to never-smokers or when the first 5–15 years of follow-up or deaths from respiratory diseases are excluded. The lowest risk of all-cause mortality in the PSC dose-response analysis was at BMIs of 22–23 kg/m^2^ [22], higher than the 20–21 kg/m^2^ suggested by APCSC for IHD and diabetes [13]. All-cause as well as CVD and cancer mortality risk was lowest at 20–22.4 kg/m^2^ in the National Cancer Institute Cohort Consortium when the first 15 years of follow-up and ever-smokers were excluded (noting that all but one cohort had used self-reported weight and height) [14]. Finally, for FPG, the ERFC analysis indicated a lowest risk of CHD at levels between 4.9 and 5.3 mmol/L [18].

To use this new evidence and to reflect the uncertainties in the TMRED, we have selected the following ranges for TMRED mean (SD): 110–115 (4–6) mmHg for SBP, 3.8–4.0 (0.5–0.65) mmol/L for TC, 21–23 (1.1–1.8) kg/m^2^ for BMI and 4.9–5.3 (0.4–0.6) mmol/L for FPG. These TMREDs reflect the evidence summarized above and the empirically observed low ranges in some populations while avoiding exposing a large proportion of the population to increased risk of mortality (e.g. from haemorrhagic stroke for TC or from diseases affected by underweight for BMI).

## Discussion

Randomized trials and observational studies provide strong evidence on etiologic effects of metabolic risk factors on CVD incidence and mortality. Our results summarize the evidence on the magnitude of these effects from large cohort pooling projects from different regions of the world and provide consistent, comparable age-specific estimates of effect sizes. We found that for the four selected risk factors, proportional effects declined with age, while being generally consistent for Western vs. Asian populations; key exceptions were effects of BMI on diabetes and HHD. These estimates are essential to estimate global, regional and national disease burden that is attributable to these risk factors and inform clinical decisions and public health policies.

In calculating disease burden attributable to risk factors, risk factor exposure data must be measured or estimated for the population of interest and is likely to vary geographically and over time. On the other hand, effect sizes are often derived from epidemiological studies conducted in a different population for two reasons: First, well-designed epidemiological studies can provide unbiased estimates for the causal effects of risk factors that reflect the underlying biological relationships and tend to be generalizable to other populations. Second, it would be prohibitively costly to conduct high-quality epidemiological studies to estimate effect sizes locally for each risk assessment analysis. With an increasing number of high-quality epidemiological studies being published, there is also a need to decide whether to use effect sizes from individual studies, e.g. those conducted in populations more similar to the risk assessment population, or to pool several studies. The former approach would preserve the potentially real differences in effect size across populations. On the other hand, effect sizes from individual studies are affected by sampling variability, motivating pooling of estimates across several studies [46] similar to our approach in this analysis.

Our analysis has several strengths: we estimated age-specific RRs accounting for the age pattern of RRs using consistent and comparable methods; we included recent pooling studies in at least two regions for most risk factor-disease pairs; we conducted re-analysis of previous pooling studies to increase comparability in relation to age groups and adjustment for regression dilution bias; we quantified uncertainty incorporating both the sampling variability of the RRs from each cohort pooling study and the uncertainty due to interpolating or extrapolating RRs into consistent age groups.

These results should also be interpreted with some limitations in mind. The pooling studies used in our analysis only covered cohorts from North America, Western Europe and the Asia-Pacific. The recently reported prospective cohort studies collaboration in South Asia [15] could not be used because it has so far not reported effect sizes for the specific diseases analyzed here. The appropriate balance between new observational studies that inform risk factor effect sizes vs. evaluating known risk factor interventions in developing countries [47], [48] may be debated. While we attempted to use sources that had pooled distinct cohorts, some cohorts were included in more than one pooling project. Further, despite our efforts to pool effect sizes for disease outcomes that had the same definitions and measurements, some differences remained. Specifically, the effect of BMI on diabetes in PSC was estimated using diabetes deaths as the outcome whereas in APCSC the outcome was diabetes incidence.

We pooled evidence on the CVD effects of risk factors from observational studies. Therefore, unmeasured and residual confounding cannot be ruled out. This is less of a concern for SBP and TC where there is overwhelming evidence from randomized trials of antihypertensives and cholesterol-lowering drugs that corroborate the evidence from observational studies on causal effects and their magnitude [8], [31], [32]. For BMI and FPG, confounding remains a concern as evidence from randomized trials of disease outcomes is either very limited for practical reasons (BMI) or provides mixed results (FPG) [49]–[52]. The biological plausibility of a causal role for BMI is supported by the effects observed in trials of bariatric surgery on mediators such as SBP, TC and FPG [53], [54] and results of bariatric surgery on cardiovascular events in severely obese patients [55]. Several meta-analyses of randomized trials of intensive versus moderate glucose lowering in diabetic patients have shown significant reduction in the risk of myocardial infarction and other major cardiovascular events [56]–[58]. In particular, a meta-analysis of the 4 largest randomized trials concluded that more intensive glucose lowering causes a “modest but significant cardiovascular benefit in the short to medium term” [57]. For these reasons, and considering the overwhelming evidence from observational studies of the graded increase in risk of CVD with higher blood glucose levels, we included IHD and stroke as outcomes of high blood glucose. However, some recent randomized trials have failed to show a significant beneficial effect of intensive glucose lowering in diabetic patients on CVD mortality, possibly because of relatively old age and frailty of participants, long duration of diabetes at baseline and high prevalence of existing atherosclerotic disease at trial entry as well as lower incidence of CVD in trial populations due to concurrent treatment with statins, aspirin and antihypertensives which reduced the power of the trials to detect an effect [59]. Another issue is that some trials have been of short duration, perhaps too short to have observed an effect [60].

Recent analyses of national and regional trends in exposure to CVD risk factors have shown considerable worldwide increases in BMI [26] and blood glucose [24], concurrent with increases in SBP and TC in some regions [23], [25]. Such trends will result in substantial CVD burden in developing countries and economies in transition in the near future. Periodic and consistent monitoring of trends and the effects of these risk factors on disease burden is needed in prioritizing prevention programs. Our results provide robust, comparable, quantitative estimates of the effects of major metabolic risk factors on CVD and diabetes and are essential for informing health policies, setting prevention priorities, and estimating disease burden attributable to these risk factors.

## Supporting Information

Table S1 **Log relative risks in original age groups as reported in pooling projects.**
(PDF)
