# Supplementary material for: The Age-Specific Quantitative Effects of Metabolic Risk Factors on Cardiovascular Diseases and Diabetes: A Pooled Analysis
Source: PLoS One. 2013 Jul 30;8(7):e65174. doi: 10.1371/journal.pone.0065174 (PMC3728292; doi:10.1371/journal.pone.0065174)
Supplement: Table S1 — Log relative risks in original age groups as reported in pooling projects. (PDF) [file pone.0065174.s001.pdf]

Table S1. Log relative risks in original age groups as reported in pooling projects

Disease outcome	Original source of RRs	Original age range or age at event	Log(RR) (95% CI)
**Systolic blood pressure (SBP)**			
Ischemic heart disease	PSC	40-49	0.36(0.32-0.40)
Ischemic heart disease	PSC	50-59	0.35(0.33-0.36)
Ischemic heart disease	PSC	60-69	0.31(0.30-0.32)
Ischemic heart disease	PSC	60-79	0.26(0.25-0.27)
Ischemic heart disease	PSC	80-89	0.20(0.18-0.22)
Ischemic heart disease	APCSC	50-59	0.51(0.42-0.60)
Ischemic heart disease	APCSC	60-69	0.33(0.26-0.39)
Ischemic heart disease	APCSC	70-79	0.27(0.21-0.33)
Ischemic stroke	PSC	50-59	0.52(0.45-0.62)
Ischemic stroke	PSC	60-69	0.40(0.34-0.45)
Ischemic stroke	PSC	70-79	0.35(0.30-0.40)
Ischemic stroke	PSC	80-89	0.13(0.07-0.20)
Ischemic stroke	APCSC	50-59	0.56(0.49-0.63)
Ischemic stroke	APCSC	60-69	0.43(0.37-0.49)
Ischemic stroke	APCSC	70-79	0.33(0.26-0.40)
Ischemic stroke	APCSC	80-89	0.24(0.12-0.36)
Haemorrhagic stroke	PSC	50-59	0.48(0.42-0.54)
Haemorrhagic stroke	PSC	60-69	0.42(0.38-0.47)
Haemorrhagic stroke	PSC	70-79	0.34(0.29-0.38)
Haemorrhagic stroke	PSC	80-89	0.34(0.26-0.42)
Haemorrhagic stroke	APCSC	50-59	0.76(0.69-0.82)
Haemorrhagic stroke	APCSC	60-69	0.51(0.46-0.58)
Haemorrhagic stroke	APCSC	70-79	0.34(0.26-0.42)
Haemorrhagic stroke	APCSC	80-89	0.13(-0.01-0.29)
Hypertensive heart disease	PSC	68	0.76(0.69-0.80)
Hypertensive heart disease	APCSC	30-44	1.83(0.99-2.66)
Hypertensive heart disease	APCSC	45-60	0.92(0.40-1.43)
Hypertensive heart disease	APCSC	60-70	0.56(0.34-0.78)
Hypertensive heart disease	APCSC	70-80	0.43(0.21-0.63)
Hypertensive heart disease	APCSC	80-100	0.46(0.21-0.69)
Aortic aneurysm	PSC	68	0.30(0.24-0.36)
Rheumatic heart disease	PSC	64	0.15(0.06-0.25)
Inflammatory heart disease	PSC	67	0.23(0.14-0.32)
Other cardiovascular disease	PSC	65	0.30(0.17-0.41)
Other cardiovascular disease	APCSC	71	0.25(0.20-0.30)

Disease outcome	Original source of RRs	Original age range or age at event	Log(RR) (95% CI)
**Total cholesterol (TC)**			
Ischemic stroke	PSC	40-59	0.31(0.14-0.49)
Ischemic stroke	PSC	60-69	0.12(-0.01-0.24)
Ischemic stroke	PSC	70-79	-0.06(-0.16-0.05)
Ischemic stroke	PSC	80-89	-0.09(-0.23-0.05)
Ischemic stroke	APCSC	25-34	0.05(-0.37-0.46)
Ischemic stroke	APCSC	35-44	0.59(0.51-0.66)
Ischemic stroke	APCSC	45-54	0.35(0.30-0.40)
Ischemic stroke	APCSC	55-64	0.18(0.13-0.23)
Ischemic stroke	APCSC	65-74	0.10(0.03-0.16)
Ischemic stroke	APCSC	75-84	0.13(0.02-0.24)
Ischemic stroke	APCSC	85-99	0.12(-0.20-0.45)
Ischemic heart disease	PSC	40-49	0.82(0.73-0.87)
Ischemic heart disease	PSC	50-59	0.54(0.49-0.58)
Ischemic heart disease	PSC	60-69	0.33(0.30-0.37)
Ischemic heart disease	PSC	70-79	0.20(0.16-0.22)
Ischemic heart disease	PSC	80-89	0.16(0.12-0.20)
Ischemic heart disease	APCSC	25-34	0.61(0.48-0.74)
Ischemic heart disease	APCSC	35-44	0.58(0.53-0.63)
Ischemic heart disease	APCSC	45-54	0.53(0.49-0.56)
Ischemic heart disease	APCSC	55-64	0.31(0.28-0.34)
Ischemic heart disease	APCSC	65-74	0.22(0.19-0.26)
Ischemic heart disease	APCSC	75-84	0.14(0.07-0.21)
Ischemic heart disease	APCSC	85-99	0.36(0.22-0.49)

Disease outcome	Original source of RRs	Original age range or age at event	Log(RR) (95% CI)
**Fasting plasma glucose (FPG)**			
Total stroke	APCSC	30-60	0.31(0.01-0.60)
Total stroke	APCSC	60-69	0.25(0.15-0.35)
Total stroke	APCSC	70-99	0.08(-0.04-0.19)
Total stroke	DECODE	25-44	-0.54(-1.31-0.22)
Total stroke	DECODE	45-54	0.17(0.04-0.30)
Total stroke	DECODE	55-64	0.18(0.11-0.25)
Total stroke	DECODE	65-74	0.17(0.10-0.24)
Total stroke	DECODE	75-99	-0.04(-0.46-0.27)
Total stroke	ERFC	63	0.12(0.04-0.22)
Total stroke	ERFC	72.8	0.13(0.07-0.21)
Total stroke	ERFC	81.8	0.07(0.07-0.19)
Ischemic heart disease	APCSC	30-60	0.35(0.23-0.48)
Ischemic heart disease	APCSC	60-69	0.18(0.06-0.30)
Ischemic heart disease	APCSC	70-99	0.18(0.07-0.29)
Ischemic heart disease	DECODE	25-44	-0.09(-0.46-0.27)
Ischemic heart disease	DECODE	45-54	0.18(0.10-0.25)
Ischemic heart disease	DECODE	55-64	0.17(0.13-0.19)
Ischemic heart disease	DECODE	65-74	0.13(0.09-0.17)
Ischemic heart disease	DECODE	75-99	0.14(0.06-0.23)
Ischemic heart disease	ERFC	62.8	0.12(0.08-0.17)
Ischemic heart disease	ERFC	72.5	0.14(0.08-0.21)
Ischemic heart disease	ERFC	81.8	0.10(0.05-0.17)

Disease outcome	Original source of RRs	Original age range or age at event	Log(RR) (95% CI)
		**Body mass index (BMI)**	
Ischemic heart disease	PSC	35-59	0.41(0.33-0.48)
Ischemic heart disease	PSC	60-69	0.34(0.28-0.40)
Ischemic heart disease	PSC	70-79	0.27(0.21-0.34)
Ischemic heart disease	PSC	80-89	0.26(0.16-0.37)
Ischemic heart disease	ERFC	64.5	0.34(0.30-0.39)
Ischemic heart disease	ERFC	75.1	0.26(0.21-0.31)
Ischemic heart disease	ERFC	85.5	0.20(0.14-0.26)
Ischemic heart disease	APCSC	30-59	0.59(0.47-0.69)
Ischemic heart disease	APCSC	60-69	0.26(0.13-0.38)
Ischemic heart disease	APCSC	70-99	0.15(0.08-0.24)
Ischemic stroke	PSC	71.8	0.32(0.21-0.44)
Ischemic stroke	APCSC	30-59	0.56(0.41-0.69)
Ischemic stroke	APCSC	60-69	0.29(0.13-0.47)
Ischemic stroke	APCSC	70-99	0.13(0.01-0.26)
Ischemic stroke	ERFC	65.9	0.34(0.27-0.41)
Ischemic stroke	ERFC	76.6	0.18(0.10-0.26)
Ischemic stroke	ERFC	86.5	0.13(0.02-0.23)
Haemorrhagic stroke	PSC	66.2	0.42(0.28-0.58)
Haemorrhagic stroke	ERFC	66	0.36(0.22-0.52)
Haemorrhagic stroke	ERFC	76.6	0.22(-0.04-0.46)
Haemorrhagic stroke	ERFC	86.6	0.11(-0.20-0.41)
Diabetes	PSC	35-59	1.53(1.26-1.78)
Diabetes	PSC	60-69	1.08(0.89-1.26)
Diabetes	PSC	70-79	0.77(0.64-0.90)
Diabetes	PSC	80-89	0.26(0.21-0.30)
Diabetes	APCSC	30-59	0.93(0.72-1.16)
Diabetes	APCSC	60-69	0.72(0.53-0.93)
Diabetes	APCSC	70-99	0.53(0.29-0.75)
